# 3D printed self-driven thumb-sized motors for *in-situ* underwater pollutant remediation

**DOI:** 10.1038/srep41169

**Published:** 2017-02-16

**Authors:** Fen Yu, Qipeng Hu, Lina Dong, Xiao Cui, Tingtao Chen, Hongbo Xin, Miaoxing Liu, Chaowen Xue, Xiangwei Song, Fanrong Ai, Ting Li, Xiaolei Wang

**Affiliations:** 1 College of Chemistry, NanChang University, NanChang, Jiangxi, 330031, P. R. China; 2 Institute of Translational Medicine, Nanchang University, Nanchang, Jiangxi, 330088, P. R. China

## Abstract

Green fuel-driven thumb sized motors (TSM) were designed and optimized by 3D printing to explore their *in-situ* remediation applications in rare studied underwater area. Combined with areogel processing and specialized bacteria domestication, each tiny TSM could realize large area pollutant treatment precisely in an impressive half-automatically manner.

One of the most pervasive problems throughout the world is inadequate access to clean water^1^. There are at least 1.1 billion people could not get safe water^2^ and resulted 50 million deaths annually because of the water-related diseases in the world^3^. A variety of methods have been used to solve this problem, such as chemical method^4^, biosorption^4^,^5^,^6^ activated sludge technology^4^,^7^, biochemical methods^8^,^9^, Fenton oxidation process^10^,^11^, and electrochemical remediation technologies^12^,^13^. Unfortunately, very few of which involved *in situ* underwater pollutant treatment^14^. From 2010 to 2016, there are about 150,000 papers on water treatment, less than 50 of which involved underwater areas. This is mainly because the lack of a cost-effective vehicle that can reach underwater pollutants^15^. Furthermore, even if the underwater vehicle has identified underwater pollutants successfully, it is still difficult to effectively deal with large areas of contamination due to the limited space of the vehicle itself. Appropriate protocols thus also needed when a large expanse of underwater pollutants was found by the underwater vehicle^16^,^17^.

Micromotors^1^ are currently a research area of intense activity due to numerous potential applications, such as environmental remediation^18^,^19^, drug delivery^20^,^21^, cellular isolation^22^, microsurgery^23^, and bioimaging^24^. However, most of these micromotors require H_2_O_2_ as the fuel sources^25^. Very few exceptional reports utilized water contaminant as fuel for the mircomotor^26^. And the size was usually too small to guarantee the cruise duration and decontamination performance^27^. These drawbacks hinder some practical applications of self-propelled micromotors in environmental monitoring and remediation.

Herein, a new water remediation method, based on self-propelled multifunctional 3D printed thumb-sized motors (TSMs), was proposed and implemented for *in-situ* underwater pollutant remediation. To guarantee the remediation efficiency, four functional units were incorporated in the TSM: green fuel for self propulsion, magnetic oxide (Fe_3_O_4_) nanoparticles for magnetic guidance, ultralight areogel^28^ for high efficient pollutant absorption, and special domesticated *B.substilis*^29^ for pollutant degradation. Figure 1 showed that the as-fabricated TSMs have a typical ellipsoidal structure with a hollow interior and two open holes in the shell. One was used for water flooding, and the bigger hole on the other side was used for drainage. Compared to the existing peroxide-driven micromotors, the present TSMs were propelled by CO_2_ bubbles, produced by the reaction of C_4_H_6_O_3_ and NaHCO_3_ in water, which were encapsulated in the inner cavity, without adding any external H_2_O_2_ fuel. It is thus more suitable for practical usage and also more environmental friendly.

The TSM was made of two identical halves; which were sealed with the organic soluble resin. As illustrated schematically in Fig. 1, in the clean water, it could swim freely, once entered the organic polluted area (such as chloroform or diethyl ether), the seal would be dissolved slowly, and resulted in continuous ejection of CO_2_ bubbles through the surrounding gap, and made it stop owing to the symmetric force consumption. By which means, after TSM has reach the polluted area, it would stop and split into two half automatically to release inner *B.substilis*. Similarly to the purification process under water, the pollutant on the surface of water also could be remedied (Figure S1).

The basic principle of self-propulsion is that bubbles were generated in the opposite direction of the motion of the TSM, which was driven by the reaction force (F) of the bubbles. The magnitude of the thrust is equal to the rate of change of momentum in the unit time of the fluid flowing through the channel of the thruster^29^.


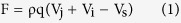



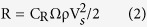


Here ρ is fluid density (kg/m^3^); q is the flow of water through the flow channel (m^3^/s); V_j_ is the velocity of bubbles (m/s); V_i_ is the velocity of water outflow; V_s_ is the velocity of TSM (m/s); R is the resistance of advanced; C_R_ is the resistance coefficient; Ω is wetted surface area (m^2^), Ω = π D L, D is diameter of TSM (m), L is the length of TSM (m). When F ≧ R, the TSM would moved forward under the action of thrust. The inlet was located to the head of TSM, water flow through the water inlet channel to reach the outlet channel, finally outflow from the outlet. According to the principle of momentum theorem and Bernoulli’s equation^30^,^31^, the flow of water from the outlet can also produce a thrust.

According to the above equations, the movement of the TSM can be tailored by several parameters, including external shape and internal structure; the speed of the flow and the velocity of the bubble. For instance, the velocity of the TSM can be improved by the optimization of streamlined shape^32^. On the other hand, their cruise duration is dependent on the drug loading capacity. Figure 2(b,e) shows the autonomous motions of four different 3D designed TSM in the water. A long tail of CO_2_ bubbles were ejected from water outlet of each TSM. These bubbles would engender a strong momentum and propelled the TSM forward with a remarkable average speed of 1.29 ± 0.04 cm/s, as summarized in the Table S1 (Movie S1). These different types of design had different velocity and cruise duration. Designs (T1, T2) with superior stream line possessed superior speed. Because design (T3) owned many small holes, which could enhance the reaction of the fuel with water, therefore, it could swim the fastest. On the other hand, due to the quick fuel consumption, this design (T3) couldn’t sustain a long time. The shape of design (T4) was wider, which increased the resistance. Its internal structure was also more complex which heavier the body weight, thus the speed was slowed down naturally. Similarly, the effects of bacteria release from TSMs are showed in Figure S2, they mainly depend on the design. As could be seen from the Table S1, design (T1) has the best overall performance compared to others. Hence, it was chosen in the subsequent study for microbial transport. Attributing to the Fe_3_O_4_, the as-obtained TSMs have a good response to the external magnetic field, and their motion could be modulated magnetically. Meanwhile, they could realize the movement way of “stop-and-go” or “stop-turn-and-go” by adjusting the magnetic field direction. As illustrated in Fig. 2g and h, to acquire the track lines of the TSMs movement, four different colored dyes were mixed with the fuel of each TSM respectively. When the fuel reacted with water, these dyes would overflow with the ejected bubbles, subsequently formed visible track lines. With the aid of magnetic field, they could move in a straight line (Fig. 2h).

Considering the print accuracy, currently, Stereo lithography technology (SLA) was chose to build TSM. Admittedly, there is a slight environmental pollution problems existed in photosensitive resin. Accidentally, we found those photosensitive resins formed TSMs were lipophilicity, and could selectively adsorb oil from water. Therefore, in some cases, they could be directly used for the small area oil remediation without any further surface modification. As shown in Figure S3(a–d), the TSM is hydrophobic, but could absorb oil quickly. As illustrated in Figure S3e, with the aid of magnetic guidance, such TSMs could perform “on-the-fly” action to pick up oil droplets. Early researches have been reported the surfaces with hydrophobic properties would hold considerable promise for removal the oil droplets and isolating other hydrophobic targets form waste water samples^33^,^34^.

However, the wastewater treatment efficiency was still limited by using the lipophilicity of photosensitive resin alone. More efficient materials are needed to improve the practical absorption performance of TSM, especially in the underwater area. Considering the limited load capacity of the TSM, this auxiliary material was expected to have both properties of low-density and high absorption capacity. Ultra light porous structure of aerogel, as one of the hotspots in the current material research, was thus synthesized and utilized for the subsequent study to enhance the adsorption efficiency. Scanning electron microscopy (SEM) depicts the porous structure of the jackfruit-aerogel (Fig. 3a) used in this study. According to the absorption result, this natural derived areogel could absorb over 400 times than its own weight out of oil, 1132 times diethyl ether, and 1375 times chloroform (Figure S4). Besides, it is worth mentioning that, after pre-treated with the target reagent, this aerogel would exhibit impressive repellence to other liquid. The selective adsorption ability of TSM was thus significantly enhanced by this interesting “memory” adsorption characteristic, which might had a great significance to remove pollutants precisely in water. To confirm this capacity, chloroform and diethyl ether were chosen for experiment. At first, aerogel was pre-treated with chloroform, for the purpose of selective adsorption of chloroform in underwater area. As depicted in Fig. 3c, the different absorbing effect of areogel toward different solvents (chloroform, diethyl ether and water) could be observed visually. This aerogel had good adsorption capacity of chloroform; however, the absorption of water and diethyl ether was much less. Subsequently, underwater experiment was also conducted by such TSMs. In the presence of chloroform, water and diethyl ether, such TSMs would only absorb chloroform, and avoid water or diethyl ether adsorption (Fig. 3b,c). The relative video information was provided in the supporting information (Movie S2, S3).

Apart from pollutant absorption, it would be more meaningful if the TSM could offer *in situ* remediation directly in the underwater environment. The entire design of TSM was compact and tiny to realize a quick and convenient usage. However, this characteristic also brought problems in the limited amount of drug loading. If the carrier is conventional water treatment agents (active carbon, quartz sand, polymeric aluminum chloride, etc.), it’s obviously difficult to effectively deal with a large area of water pollutants. Therefore, a “self-proliferated agent” concept was selected here, which was realized by using microbial degradation^35^,^36^. Herein, *B.substilis* was selected as the loading microbe that used for *in situ* water remediation. This strain can quickly dominant the underwater environment and effectively degrade petroleum-oil^37^, sulfur, nitrogen and chlorine pollutants^38^. What’s more, *B.substilis* would not damage the human body, but even regulate the intestinal health of people by inhibiting the growth of pathogenic bacteria^39^. It is thus an ideal selection for the purpose of environmental safety. However, the original strain of *B.substilis* did not own enough degradation efficiency. To get more specialized strain, the experiment of screening and domestication was carried out. The results of biodegradability (Fig. 4) demonstrated that, the original strain were 70.43%, which was lower than domesticated strain (76.82%). More importantly, to achieve this effect, the original strain must be taken almost a month, while the domesticated strain just needed less than one third of that time. Three days at the beginning, two groups of rhodamine-B were degraded to 12.38% and 64.41% treat by original and domesticated strain respectively, and the control group had not significant change. In spite of the load amounts is limited, this self-proliferated “agents” could degrade more than ten thousand times pollutants compared to its initial loading volume. As shown in Figure S5, after seven consecutive days of degradation, the obvious color fading could be seen. Herein, the volume of bacteria liquid was 250 μL, while the pollutants volume was 3000 mL.

In this study, we have presented the first example of self-propelled 3D printing TSM for *in-situ* underwater remediation. Instead of traditional H_2_O_2_ fuel, such TSMs were propelled by the CO_2_ bubbles, which were generated by green chemical reagents — C_4_H_6_O_3_ and NaHCO_3_. The new green-driven motion capability would greatly enlarge the application fields, where the use of the traditional peroxide fuel is not convenient or safety. Incorporated with ultralight functional material and microbial acclimation, such 3D printed TSMs, though tiny and compact, have integrated two core capabilities for *in-situ* underwater remediation: high-efficient selective adsorption, and huge amount degradation. On the current stage, this technology is still in infancy. There are a lot of room for improvement, such as the cruising range; a practical feasible magnetic guidance manner; and how to deal with the pollution of complex components. With the advancement of material science and 3D printing technology, the above problems are expected to be solved gradually. In particularly, we expect to narrow the size of this functional TSM down to the micron level. By that time, these green-driven micromotors might not only apply in underwater area, but also have profound influences on diverse biomedical or industrial applications, such as biological targets isolation, *in-vivo* targeted drug delivery and anti-thrombotic therapy.

## Methods

### Fabrication of TSM

TSMs with ellipsoidal-like structure were fabricated by FSL3D Pegasus Touch 3D printer. The TSM was designed by Rhinoceros.

### Preparation of aerogels

Taking fresh jackfruit, removed the cores, the flesh were put into reaction kettle. Reaction kettle was transferred to a tubular furnace for pyrolysis, and then was heated to 180 °C for 12 h. After that, the preliminary aerogel was immerged in absolute ethyl alcohol, until the solution became colorless. Subsequently, this aerogel was put into water to remove the ethyl alcohol. Next, the aerogel was pre-freezed at −180 °C for 24 h and freeze-dried 12 h in freeze dryer. Finally, the aerogel was obtained.

## Additional Information

**How to cite this article:** Yu, F. *et al*. 3D printed self-driven thumb-sized motors for *in-situ* underwater pollutant remediation. *Sci. Rep.* **7**, 41169; doi: 10.1038/srep41169 (2017).

**Publisher's note:** Springer Nature remains neutral with regard to jurisdictional claims in published maps and institutional affiliations.

## Supplementary Material

Supplementary Movie S1

Supplementary Movie S2

Supplementary Movie S3

Supplementary Movie S4

Supplementary Movie S5

Supplementary Information

## Figures and Tables

**Figure 1:**
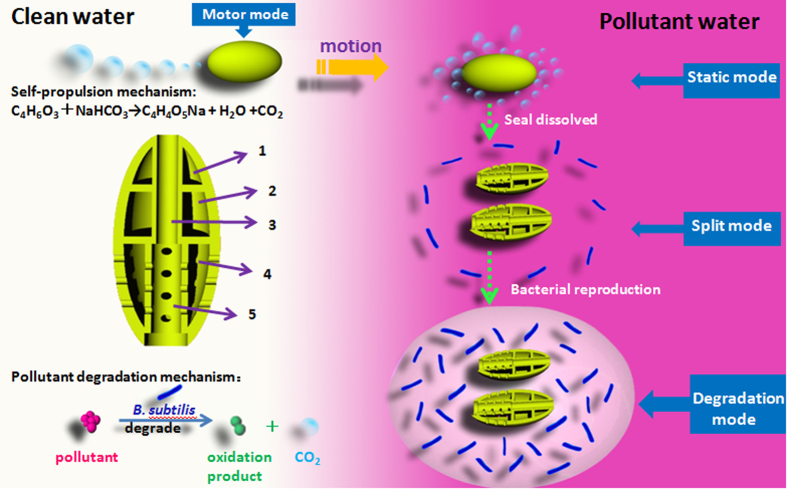
Schematic representation of the self-propulsion of micromotor and the process of degradation of organic contaminant by microbe. The micromotor includes a water inlet, water outlet, and three grooves, which are used for encapsulating Fe_3_O_4_ nanoparticles, *B.substilis* and fuel, respectively.

**Figure 2:**
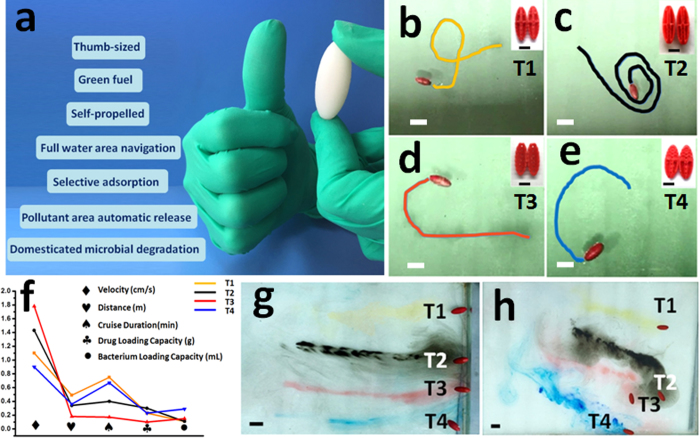
Motion of TSMs in water. (**a**) Thumb-sized motor with a series of advantage. (**b**,**c**,**d**,**e**) autonomous motion with four types of TSM and their movements, scale bar is 3.9 cm. The inserts are internal structure images of each TSM; scale bar is 1 cm. (**f**) Different performance parameters of these four type’s TSM, respectively are velocity, drug loading capacity, bacterium loading capacity, distance and cruise duration. (**g**,**h**) Optical photos of four colors motion curve of four different types’ TSM without external magnetic field (**g**) and in an external magnetic field (**h**); scale bar is 3.9 cm.

**Figure 3:**
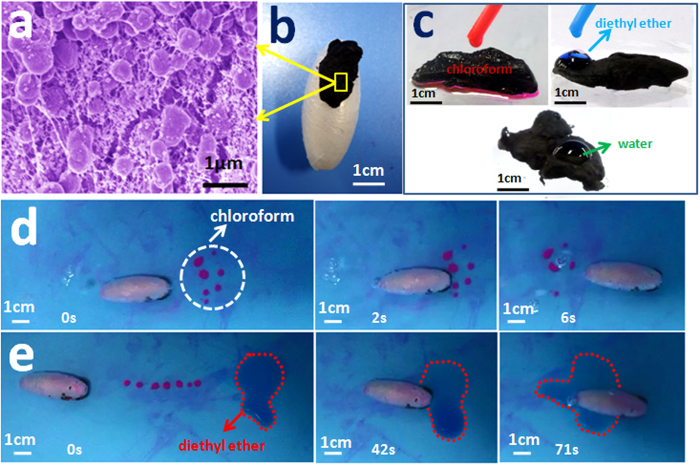
TSMs with aerogel have the ability to selective adsorbed organics. The pink drops are chloroform, the blue drop is diethyl ether, and the colorless drop is water. (**a**) Scanning electron microscopy (SEM) image of aerogel; (**b**) optical photograph of TSM with aerogel; (**c**) optical photographs of different adsorption effects of aerogel to chloroform (red), diethyl ether (blue) and water (colorless). (**d**,**e**) “On-the-fly” selective adsorption of chloroform used the TSM which stick equipped with aerogel under water.

**Figure 4:**
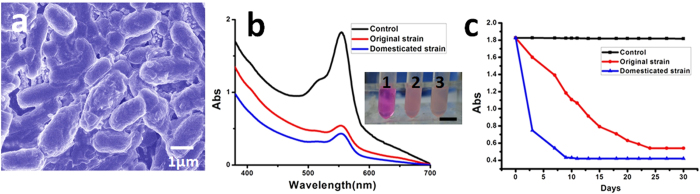
Degradation of rhodamine-B by *B.substilis* (domesticated strain and original strain). (**a**) Scanning electron microscopy (SEM) image of *B.substilis*. (**b**) UV-visible absorption spectra with different treatments. The insert is optical graph of rhodamine-B after treated with 1: control, 2: original strain and 3: domesticated strain. Scale bar is 1 cm. (**c**) The relative degradation curve with time.
