# Supplementary material for: 3D printed self-driven thumb-sized motors for in-situ underwater pollutant remediation
Source: Sci Rep. 2017 Feb 16;7:41169. doi: 10.1038/srep41169 (PMC5311938; doi:10.1038/srep41169)
Supplement: Supplementary Information [file srep41169-s6.pdf]

Supplementary Information

3D printed self-driven thumb-sized motors for in-situ underwater pollutant remediation

Fen Yu[1], Qipeng Hu[2], Lina Dong[2], Xiao Cui[2], Tingtao Chen[2], Hongbo Xin[2], Miaoxing Liu[1], Chaowen Xue[2], Xiangwei Song[1], Fanrong Ai[2], Ting Li[2], Xiaolei Wang[1,2]*

(1) College of Chemistry, NanChang University, NanChang, Jiangxi, 330031, (P.R.China)

(2) Institute of Translational Medicine, Nanchang University, Nanchang, Jiangxi, 330088 (P.R.China)

Supporting Movie Legends

Movie S1. This movie shows these different types of TSM had different velocity and cruise duration.

Movie S2, S3. This movie shows TSMs with aerogel have the ability to selective adsorbed organics.

Movie S4. This movie shows TSM with magnetic guidance on water surface.

Movie S5. This movie shows TSM with magnetic guidance under water.

Experimental:

1. *Fabrication of TSM*: TSMs with ellipsoidal-like structure were fabricated by FSL3D Pegasus Touch 3D printer. The TSM was designed by Rhinoceros.
2. *Preparation of aerogels:* Taking fresh jackfruit, removed the cores, the flesh were put into reaction kettle. Reaction kettle was transferred to a tubular furnace for pyrolysis, and then was heated to 180 ℃ for 12 h. After that, the preliminary aerogel was immerged in absolute ethyl alcohol, until the solution became colorless. Subsequently, this aerogel was put into water to remove the ethyl

alcohol. Next, the aerogel was pre-freezed at -180 ℃ for 24 h and freeze-dried 12 h in freeze dryer. Finally, the aerogel was obtained.

3. *Screening and Domestication of Strains*: Pick several purified strains (*B.substilis*), put them into solution of rhodamine-B, respectively, finally observed the color of medium after static culture for 64 h at 37 ℃. If the color faded out, it would suggest that strain could degrade organism. The lighter the color showed, the stronger the ability of degradation was. Through organic decomposition test, strains which could highly degrade rhodamine-B were screened out. Subsequently, the screening strain was incubated in a mixture medium with Lysogeny broth (LB) and rhodamine-B. After several rounds of this domestication, strain with stronger degrading capability would be obtained.
4. *Degration of pollutant: B.substilis* strains which domesticated and original were put into the polluted water (rhodamine-B), respectively. That without B.substilis was control group. The amount of water and bacteria in each group were the same, and they were cultured at 37 ℃ to make the bacteria increased rapidly. The amount of rhodamine-B was measured by UV-visible absorption spectra, every once in a while.

Table S1: Statistical velocity and cruise duration

**Number**	**T1**	**T2**	**T3**	**T4**
**Velocity (cm/s)**	1.09 ± 0.02	1.43 ± 0.04	1.78 ± 0.09	0.89 ± 0.01
**Cruise duration (s)**	49	34	18	36

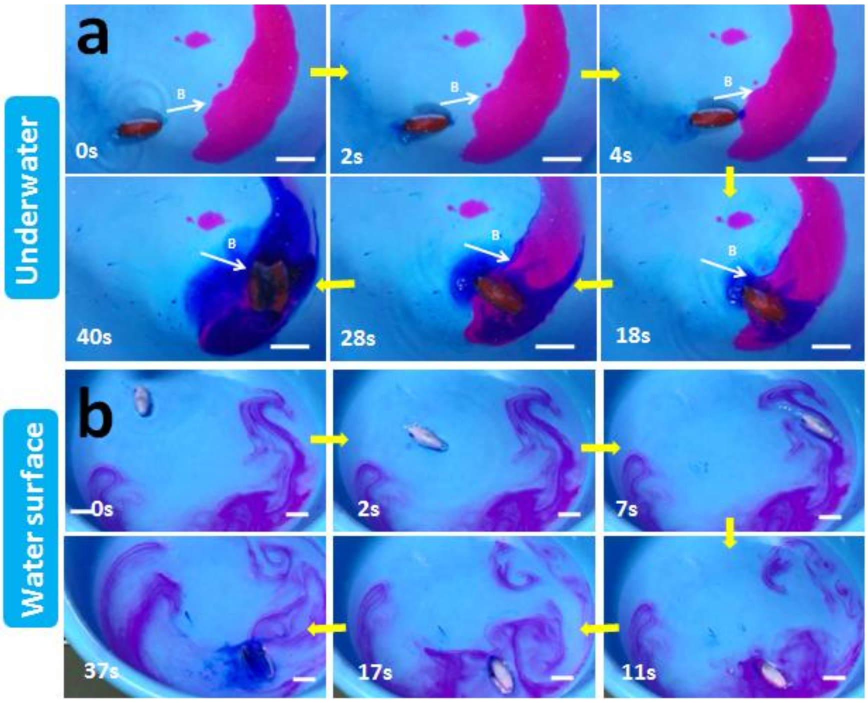

Figure S1. Time-lapse images of TSMs with magnetic guidance. (a) Under water. 0-4 s are the process of directed motion; 4-40 s are the dissolved process of seal. (b) On the water. 0-7 s are the process of motion; 11-37 s are the dissolved process of seal. Scale bar is 4cm. Blue liquid that released from TSMs was represented the microbe (*B.substilis*).

The removal of contaminant under water is of considerable importance for minimizing environmental hazards. Actually, the TSMs were found that could work under the water which just needed an extra counterweight. To confirm this, a simulated underwater environment containing pollutant was created. As shown in figure S3, with the guidance of an external magnetic field and its autonomous motion, the TSM could slowly swim under water; once entered in the place with contaminant, the seal would gradually dissolve, subsequently released microbe slowly. Finally, the TSMs would be split in half, and then the microbe would be released completely. Similarly to the purification process under water, the pollutant on the surface of water also could be remedied undoubtedly. That’s to say, whether under or on the water,

these TSMs could play a similar role, which just depended on where was the pollutant.

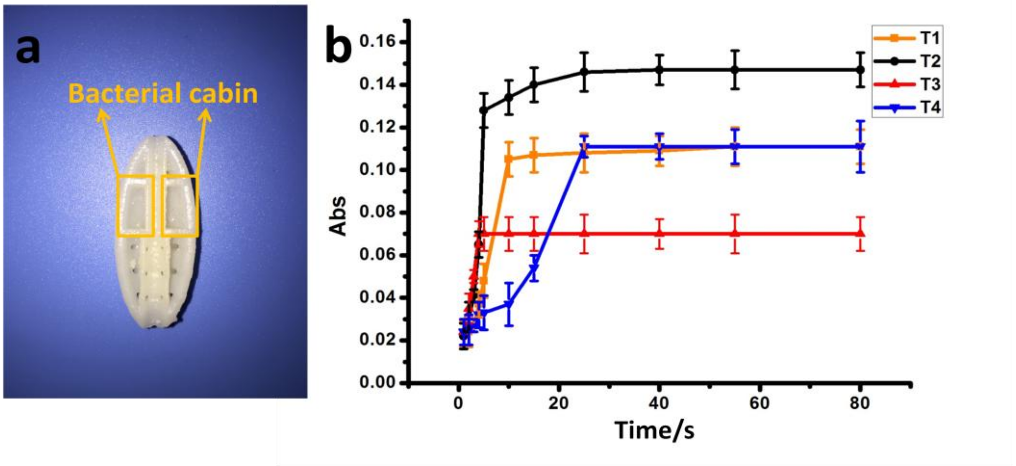

Figure S2. Effects of bacteria release. (a) Optical photograph of TSM (T1). (b) Release curve of bacteria from these four different TSMs.

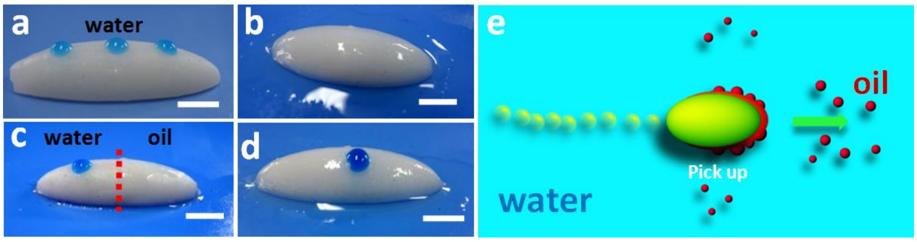

Figure S3. Oil captured using TSM. (a-d) Optical photograph of TSM surface activity to water and oil, blue drop is water, the other was oil. (e) Schematic demonstration of the bubble propulsion and the removal of oil droplets by the TSM due to the hydrophobic outer surfaces.

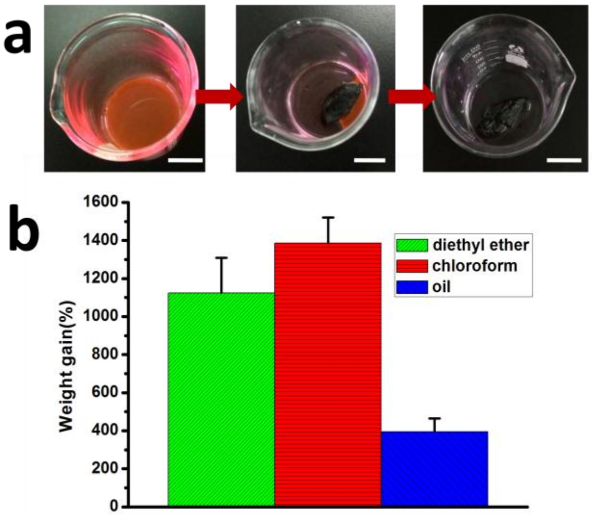

Figure S4. Absorption efficiency of the jack fruit aerogels. (a) Diethyl ether is absorbed by a jack fruit aerogel completely in 30 seconds. (b) Absorption efficiency of jack fruit aerogels for various liquids.

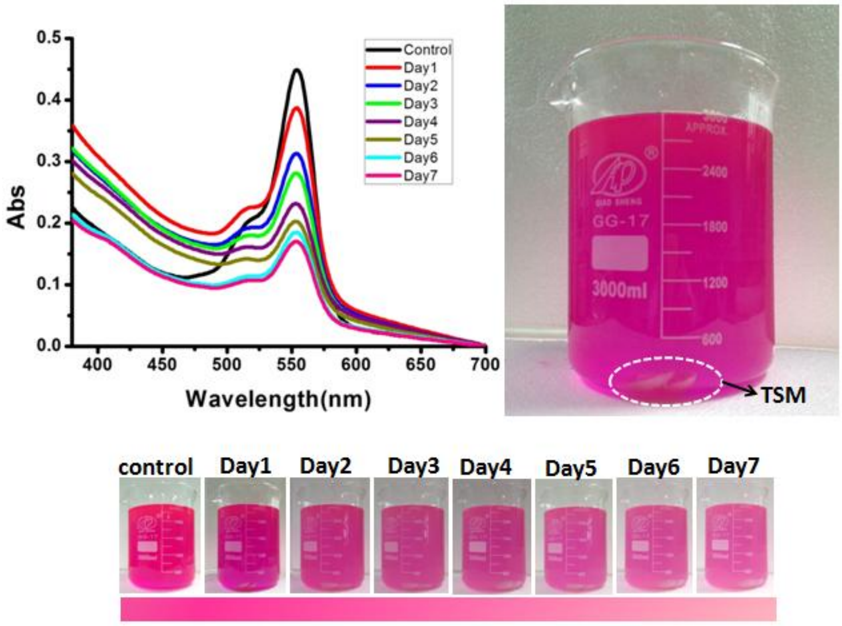

Figure S5. Degration of Rhodamine-B by domesticated *B.substilis* strain for seven days.
